# Abscopal effect of radiation on multiple lung metastases of lung adenocarcinoma: a case report

**DOI:** 10.1186/s12885-019-5566-8

**Published:** 2019-04-08

**Authors:** Aoi Kuroda, Takaya Tabuchi, Eri Iwami, Kotaro Sasahara, Tatsu Matsuzaki, Takahiro Nakajima, Yuki Tsutsumi, Keisuke Eguchi, Takeshi Terashima

**Affiliations:** 1 Department of Respiratory Medicine, Tokyo Dental College Ichikawa General Hospital, 5-11-13 Sugano, Ichikawa, Chiba 272-0824 Japan; 2 Department of Internal Medicine, Tokyo Dental College Ichikawa General Hospital, 5-11-13 Sugano, Ichikawa, Chiba 272-0824 Japan; 3 Department of Radiology, Tokyo, Dental College Ichikawa General Hospital, 5-11-13 Sugano, Ichikawa, Chiba 272-0824 Japan; 4 Department of Surgery, Tokyo Dental College Ichikawa General Hospital, 5-11-13 Sugano, Ichikawa, Chiba 272-0824 Japan

**Keywords:** Abscopal effect, Adenocarcinoma, Irradiation, Lung cancer, Pulmonary metastases

## Abstract

**Background:**

Abscopal effect is the out-of-field response to localized irradiation therapy that results in systemic antitumorigenic effects such as the regression of a tumor distant from the target site.

**Case presentation:**

A 76-year-old woman was diagnosed with pulmonary adenocarcinoma (cT1bN0M0 stage IA), and right upper lobectomy was performed in November 2015. The pathological stage was pT1bN2M0 stage IIIA. Genomic analysis revealed an *EGFR* mutation. Immunohistochemical analysis revealed a programmed death-ligand 1 tumor proportion score of < 1%. The patient was under watchful observation without adjuvant chemotherapy. Multiple mediastinal and right hilar lymph node metastases were found in February 2018. Radiation therapy at a total dose of 60.0 Gy distributed in 30 fractions was performed over a period of 6 weeks. A computed tomography (CT) scan performed 6 weeks after irradiation therapy showed a reduction in lymph node metastases. However, left hilar and right supraclavicular lymph node metastases and multiple pulmonary metastases were newly observed outside of the irradiation field. A CT scan performed 6 weeks later showed a dramatic complete disappearance of the previously observed pulmonary metastases. No chemotherapy was administered during the period.

**Conclusion:**

This was a case of abscopal effect: irradiation of the mediastinum resulted in the disappearance of multiple pulmonary metastases in both lungs.

## Background

An abscopal effect has been defined as a systemic antitumorigenic effect that results in the regression of a tumor from the irradiated site by localized irradiation therapy. The first abscopal effect was reported by Mole [1], and other cases in several tumor types, including lymphoma, melanoma, and renal cell carcinoma, have since been reported [2]. There have been, however, only a few case reports of abscopal effect in non-small cell lung cancer (NSCLC) [3–8].

Herein, we report a case of lung adenocarcinoma showing complete disappearance of multiple pulmonary metastases after mediastinal irradiation therapy, suggesting an abscopal effect.

## Case presentation

**Fig. 1:**
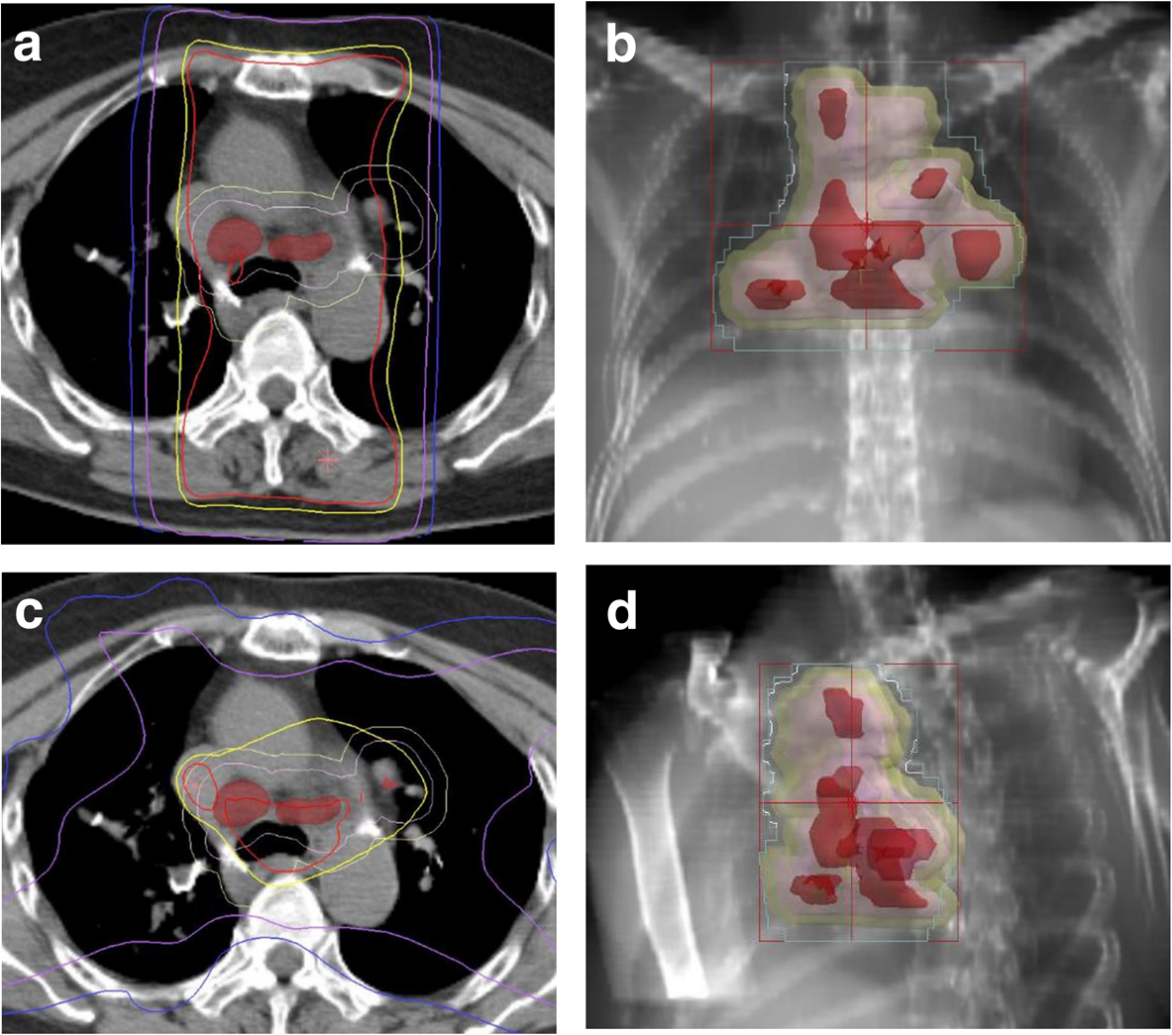
Radiation therapy to a total dose of 60.0 Gy, distributed in 30 fractions, was performed over a period of 6 weeks. In the first 4 weeks, the treatment was prescribed to the 100% isodose curve to a total dose of 40.0 Gy distributed in 20 fractions using opposing anterior-posterior fields (**a**, **b**). An additional 20.0 Gy was distributed in 10 fractions using 10 beams in the subsequent 2 weeks (**c**, **d**). The curves represent 100% isodose (red), 95% isodose (yellow), 50% isodose (purple), and 30% isodose (blue) (**a**, **c**). Gross tumor volume, clinical target volume, and planning target volume are shown in red, pink, and yellow, respectively (**b**, **d**)

**Fig. 2:**
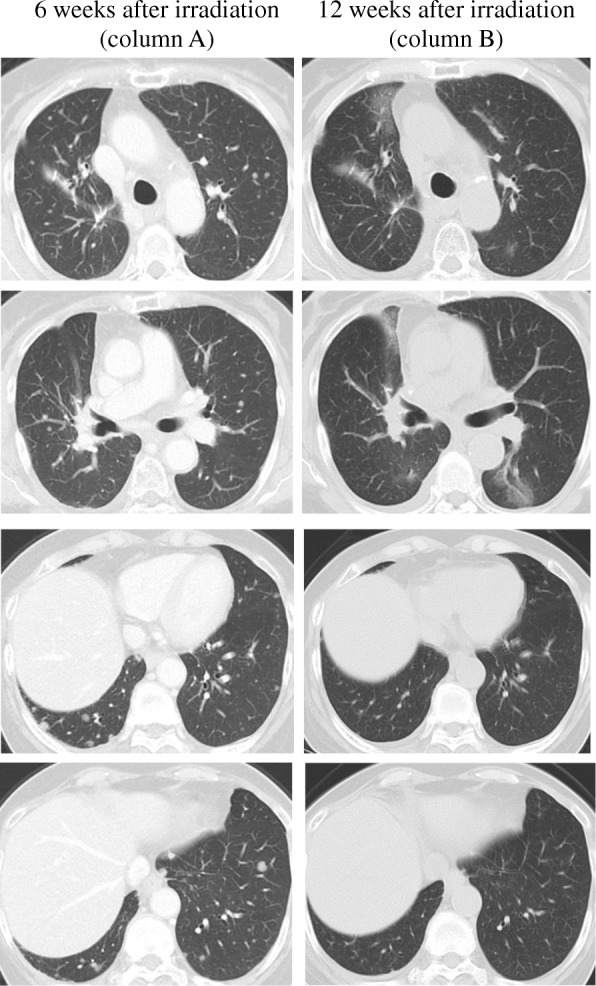
A computed tomography scan of the chest, performed 6 weeks after the irradiation therapy against multiple mediastinal and right hilar lymph node metastases, showed appearance of multiple pulmonary nodules in both lungs, suggesting metastases (column A). A follow-up computed tomography scan of the chest, performed 12 weeks after the irradiation therapy, showed complete disappearance of the multiple pulmonary metastases associated with radiation pneumonitis (column B)

**Fig. 3:**
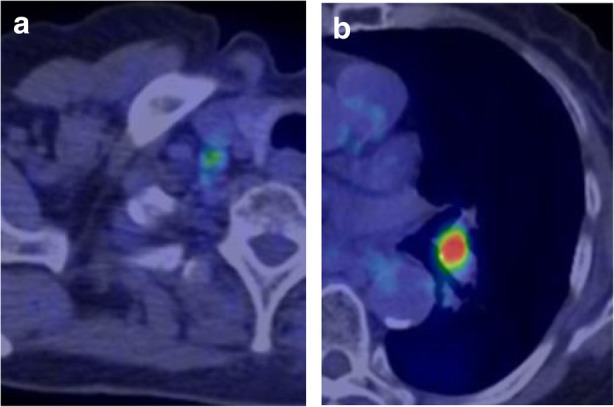
Positron-emission tomography/computed tomography scan, performed 12 weeks after the irradiation therapy, showed the uptake of 18-fluorodeoxyglucose in the right supraclavicular (**a**) and the left hilar (**b**) lymph nodes

**Fig. 4:**
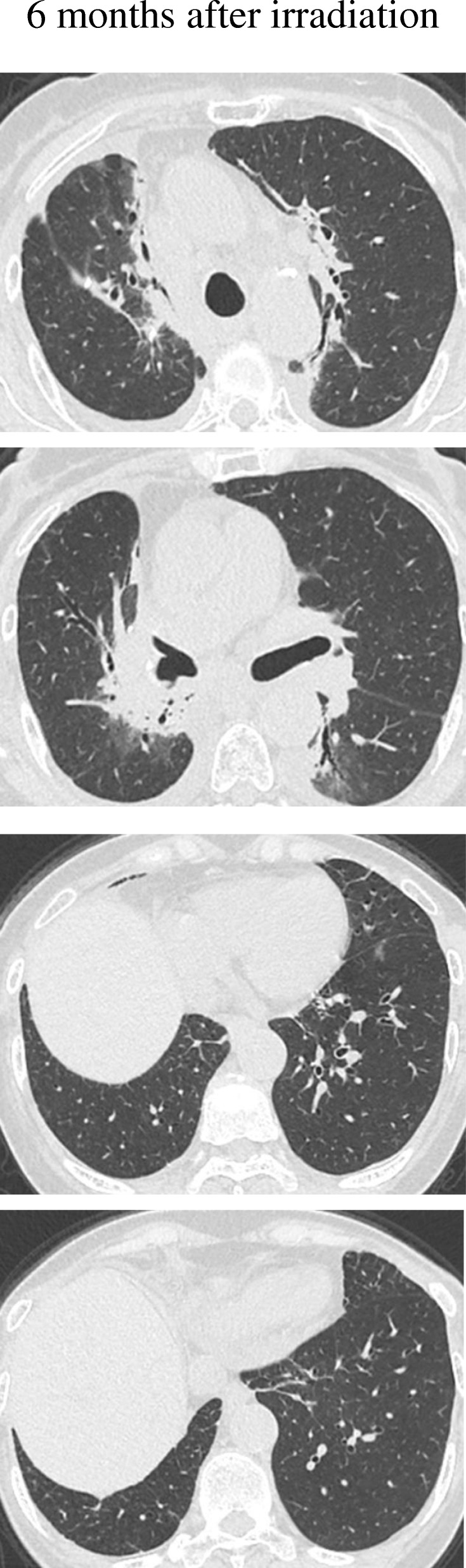
A follow-up computed tomography scan of the chest, performed 6 months after the irradiation therapy, showed radiation pneumonitis but no reappearance of pulmonary metastases in either lung

**Fig. 5:**
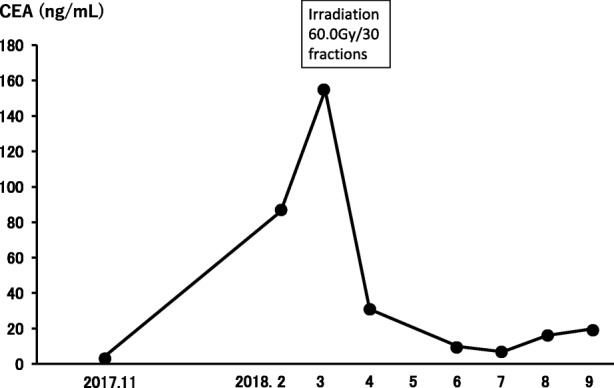
Clinical course. The levels of serum carcinoembryonic antigen (CEA) dropped from 154.5 ng/mL to 30.9 ng/mL after the irradiation therapy and further decreased to 6.8 ng/mL at 3 months post treatment; however, the levels increased again to 19.7 ng/mL at 6 months after completion of the irradiation therapy

A 76-year-old woman was referred to our hospital because of a nodule in the right upper lobe. The patient had no respiratory symptoms. The computed tomography (CT) scan of the chest revealed a 2.3 × 1.6 cm nodule, and the patient was diagnosed with pulmonary adenocarcinoma (cT1bN0M0, stage IA, according to the TNM classification of the Union for International Cancer Control (UICC), 7th edition; cT1cN0M0, stage 1A3, according to the UICC, 8th edition). The patient had no smoking history. Her comorbidities consisted of hypertension and hyperlipidemia. The right upper lobectomy was performed in November 2015, and the pathological stage was pT1bN2M0, stage IIIA. Genomic analysis revealed the epidermal growth factor receptor (*EGFR*) gene mutation L858R in exon 21. Immunohistochemical analysis revealed a programmed death-ligand 1 (PD-L1) tumor proportion score (TPS) of < 1%. The patient was under watchful observation without adjuvant chemotherapy. Multiple mediastinal and right hilar lymph node metastases were found in February 2018. Radiation therapy alone was selected because the recurrence of the disease was limited to the local region and the patient was 79 years old at the time of recurrence. Radiation therapy amounting to a total dose of 60.0 Gy, distributed in 30 fractions, was performed over a period of 6 weeks. The 3D radiotherapy-planning technique was used. In the first 4 weeks, 40.0 Gy was distributed in 20 fractions using opposing anterior-posterior fields (Fig. 1a, b). In the subsequent 2 weeks, an additional 20.0 Gy was distributed in 10 fractions using 10 beams (Fig. 1c, d) for covering multiple mediastinal lymph node metastases but sparing the spinal cord and hilum of the left lung. The planning target volume included multiple mediastinal and right hilar lymph nodes, with a 10 mm margin to account for microscopic disease, internal moving, and setup errors. The percentage volume of lung receiving a dose of more than 20 Gy (V20) was 29.3%. Static radiotherapy was delivered using a 10 MV X-ray and five fractions per week. A chest CT scan performed 6 weeks after the irradiation therapy showed reduction of the lymph node metastases. However, left hilar and right supraclavicular lymph node metastases and multiple pulmonary metastases were newly observed outside of the irradiation field (Fig. 2). EGFR tyrosine kinase inhibitor (TKI) treatment was planned as a start. Interestingly, a follow-up chest CT scan performed 12 weeks after the completion of irradiation therapy showed complete disappearance of the multiple pulmonary metastases associated with radiation pneumonitis (Fig. 2). However, there was no change in the size of the left hilar and the right supraclavicular lymph node metastases, which was confirmed by 18-fluorodeoxyglucose positron-emission tomography (PET)/CT (Fig. 3a, b). The patient was therefore under watchful observation, without receiving EGFR-TKI treatment. A follow up CT scan performed 6 months after the completion of irradiation therapy showed a slight increase in the size of the lymph node metastases, but no reappearance of multiple pulmonary metastases nodules (Fig. 4). The levels of serum carcinoembryonic antigen (CEA) dropped from 154.5 ng/mL to 30.9 ng/mL after the irradiation therapy and further decreased to 6.8 ng/mL by 3 months post irradiation therapy. The levels, however, increased again to 19.7 ng/mL at 6 months after completion of the irradiation therapy (Fig. 5). No cytotoxic chemotherapy or EGFR-TKI was given during the period. The number of CD8^+^ lymphocytes was 190/μL at 3 months and 356/μL at 4 months after irradiation.

## Discussion and conclusions

In this report, we describe an abscopal effect in a patient with NSCLC. Irradiation of the mediastinal lymph nodes resulted in the complete disappearance of multiple pulmonary metastases. The patient did not receive any other therapy during the period. Although an abscopal effect has been reported in several tumor types [2], clinical reports of it in NSCLC are very rare [3–8]. These reports included cases in which an abscopal response was observed after irradiation and during immune checkpoint inhibitor therapy [3, 4]. In another report, an abscopal response was observed after radiotherapy and during immunotherapy using dendritic cells and cytokine-induced killers in NSCLC [5]. These reports supported the hypothesis that the abscopal effect is likely mediated by the immune system. Local irradiation-induced direct tumor destruction is speculated to induce the release of circulating tumor antigens and then elicit an inflammation cascade that can activate antigen-presenting cells, which may then mediate an augmented immune response against malignant lesions that express similar tumor antigens in the out-of-target sites. Compared with previous reports in NSCLC, the case presented herein is extremely rare in the sense that no immunotherapy or immune checkpoint inhibitor was given during the course. In other words, a pure abscopal effect was documented in this case.

The present case provides important insight regarding the time when an abscopal effect occurs and for how long the effect is maintained. A literature review reported that abscopal responses occurred after 1–24 months (median 5 months), and the relapse-free time was maintained for 3–39 months (median 13 months) [9]. In the present case, the time to the documented abscopal effect was 3 months, which is consistent with the reported time; however, we could not determine the duration of the effect, because there was no reappearance of multiple pulmonary metastases nodules in spite of a slight increase in the size of lymph node metastases and in the serum CEA levels after 6 months. CD8^+^ T lymphocytes have been demonstrated to play a key role in the abscopal effect [10–12]. Systemic lymphocytopenia is known to be induced by local radiotherapy, and it usually persists for several months. It is thought that the function, rather than the number of CD8^+^ lymphocytes in peripheral blood is important in this phenomenon. On the other hand, it might be possible that the number of tumor-infiltrating CD8^+^ T lymphocytes is the most significant factor. In the present case, the number of CD8^+^ lymphocytes was low when the abscopal effect was documented and high when the regrowth of lymph node metastases and the increase in tumor marker levels was observed.

It should be emphasized that TPS was less than 1% in this case. The axis of PD-L1 and its cognate receptor programmed cell death protein 1 (PD-1) on the surface of T cells is known as a T cell-inhibiting signaling pathway. A possible explanation for the abscopal effect in the present case is that there was little inhibition by the PD-1/PD-L1 pathway on anti-tumor T cell function.

The lung adenocarcinoma in the present case harbored the EGFR mutation L858R in exon 21. There has been one case report which showed an abscopal effect in a patient with NSCLC that harbored the exon 19 deletion and exon 20 T790 M mutations [5]. Because the number of reported cases in which an abscopal effect was observed in NSCLC is extremely low, it is not possible to conclude whether there is a higher likelihood of an abscopal effect occurring in lung cancer that harbors driver mutations.

The limiting factor in this study is that no pathological confirmation of the metastatic pulmonary nodules was obtained. Moreover, it is difficult to speculate why an abscopal effect was observed in the multiple pulmonary nodules, but not the lymph node metastases. However, it is not rare that cytotoxic chemotherapy or EGFR-TKI therapy is effective in some tumor sites but not in others. Size, environment, recruitment of immune cells, and vascularity of the malignant lesion are all factors that contribute to the differences in tumor progression.

Clinical trials have shown that immunotherapy may enhance abscopal effects [13]. Recently, administration of a PD-L1 inhibitor after concurrent chemoradiotherapy for stage III NSCLC has shown a clinical effect [14]. The information obtained from our case provides important insight into when and for how long the immunotherapy should be administered to get the most benefit out of an abscopal effect.

In conclusion, we report a case of lung adenocarcinoma showing an abscopal effect resulting in complete disappearance of multiple pulmonary metastases after mediastinal irradiation therapy.

## Abbreviations

CEA: Carcinoembryonic antigen
CT: Computed tomography
EGFR: Epidermal growth factor receptor
NSCLC: Non-small cell lung cancer
PD-1: Programmed cell death protein 1
PD-L1: Programmed death-ligand 1
PET: Positron-emission tomography
TKI: Tyrosine kinase inhibitor
TPS: Tumor proportion score
UICC: Union for International Cancer Control
